# Image Quality Characteristics of Handheld Display Devices for Medical Imaging

**DOI:** 10.1371/journal.pone.0079243

**Published:** 2013-11-13

**Authors:** Asumi Yamazaki, Peter Liu, Wei-Chung Cheng, Aldo Badano

**Affiliations:** 1 Center for Devices and Radiological Health, Food and Drug Administration, Silver Spring, Maryland, United States of America; 2 Graduate School of Medical Sciences, Nagoya University, Nagoya, Aichi, Japan; University Hospitals of Geneva, Switzerland

## Abstract

Handheld devices such as mobile phones and tablet computers have become widespread with thousands of available software applications. Recently, handhelds are being proposed as part of medical imaging solutions, especially in emergency medicine, where immediate consultation is required. However, handheld devices differ significantly from medical workstation displays in terms of display characteristics. Moreover, the characteristics vary significantly among device types. We investigate the image quality characteristics of various handheld devices with respect to luminance response, spatial resolution, spatial noise, and reflectance. We show that the luminance characteristics of the handheld displays are different from those of workstation displays complying with grayscale standard target response suggesting that luminance calibration might be needed. Our results also demonstrate that the spatial characteristics of handhelds can surpass those of medical workstation displays particularly for recent generation devices. While a 5 mega-pixel monochrome workstation display has horizontal and vertical modulation transfer factors of 0.52 and 0.47 at the Nyquist frequency, the handheld displays released after 2011 can have values higher than 0.63 at the respective Nyquist frequencies. The noise power spectra for workstation displays are higher than 1.2×10^−5^ mm^2^ at 1 mm^−1^, while handheld displays have values lower than 3.7×10^−6^ mm^2^. Reflectance measurements on some of the handheld displays are consistent with measurements for workstation displays with, in some cases, low specular and diffuse reflectance coefficients. The variability of the characterization results among devices due to the different technological features indicates that image quality varies greatly among handheld display devices.

## Introduction

Rapidly advancing computer technology has succeeded in reducing the size, weight, and price of current handheld devices while improving internet accessibility and processing power. The increased popularity of mobile phones and tablet computers has made them ubiquitous in daily life as data communication tools. The use of handheld devices has also been expanding in medical imaging applications. In developing countries and rural areas, microscopy images can now be transferred to hospitals or laboratories using handheld devices for evaluation or secondary consultation [1], [2]. Handheld devices have also been shown to facilitate remote diagnosis in emergency medical care[3]–[11]. Notably, in recent years, the quality of handheld display devices has been improved with larger number of pixels, higher pixel density, and wider color gamut. However, the display characteristics differ substantially from high-resolution displays for medical workstations. Medical workstations are categorized as primary- and secondary-class displays [12]. Primary-class displays are used by radiologists and other specialists for image diagnosis. Secondary-class displays are used by other staff for reference viewing and consultation after a diagnostic report is offered. Primary-class displays should meet more strict performance criteria. For instance, the American Association of Physicists in Medicine recommends the major acceptable criteria about luminance evaluation for primary- and secondary-class displays as maximum luminance should be greater than 170 and 100 cd/m^2^, luminance ratio of maximum and minimum luminance should be greater than 250 and 100, and contrast conformance to grayscale standard display function (GSDF) should be better in 10% and 20% errors, respectively. Recent reports suggest that handheld devices can be considered alternatives to workstation devices even in the primary evaluation of medical images in radiology[6]–[11], [13].

### Previous Work

McNulty et al. investigated the diagnostic accuracy of a tablet computer (iPad 1st generation, Apple Inc., CA, USA) in comparison with a secondary-class liquid-crystal display (LCD) in the case of interpreting spinal CT and MR images in emergency examinations [6]. Thirteen American Board Radiology certificated radiologists reviewed 31 MR cases on both displays. Comparisons between data for both displays showed no statistically significant differences in terms of area-under-the-receiver-operating-characteristic-curve (AUC). Using the Dorfman-Berbaum-Mets multireader-multicase uncertainty analysis, no statistically significant difference of overall diagnostic accuracy was found between the tablet and secondary-class display. The authors concluded that tablet computers allow physicians to review MR images of spinal emergency cases with an accuracy at least equal to that of secondary-class LCDs and suggested that tablet computers can be considered useful aids in the initial image interpretation stages when secondary-class displays are not available with the added benefit of additional rapid access to drug information and medical reference programs.

Another recent paper by Christopher et al. compared diabetic retinopathy (DR) referral recommendations made by viewing fundus images using a tablet computer (iPad 1st generation) with those made using a desktop display and found that whether an expert views color fundus images on a tablet or a desktop display, their recommendations regarding DR referral are similar [13]. Using k and weighted k statistics to measure cross-platform intra-observer agreement, the study showed no significant difference between ratings corresponding to the handheld and desktop display devices. The authors concluded that tablet-based rating of color fundus images for subjects at risk for diabetic retinopathy was consistent with desktop display-based rating, indicating that tablet computers might be reliably used for clinical evaluation of fundus images.

In a recent study by John et al., tablet computers with larger screens, high-pixel count and touch screen interface were found to be advantageous compared to mobile phone devices for viewing radiological images [7]. The study assessed the potential of tablet computers to be used for emergency radiology tele-consultation by running multi-image CT and MRI studies comparing tablets to a picture archiving and communication system (PACS) workstation. The authors analyzed major findings (primary diagnosis), minor findings (incidental), and user feedback taken from readers who completed structured reporting sheets, and compared them to formal clinical reports of an initial reporting by radiologists that were retrieved from the PACS. All of the reviewing radiologists reported a favorable experience, however, it was noted that tablet computers had issues relating to software stability and some limitations due to image manipulation. Despite these shortcomings, the authors concluded that diagnosis of emergency conditions on CT and MRI using tablet computers could be made with good agreement to those reviewed on dedicated PACS workstations.

Another study by McLaughlin et al. compared a tablet computer (iPad 1*^st^* generation) with a diagnostic 2 mega-pixel monochrome LCD. Reporting discrepancies were recorded and analyzed using a web-based image interpretation system for open peer review (RADPEER) offered by the American College of Radiology. Preliminary interpretations of 100 emergency brain CT examinations on tablet computers were compared to formal review on the diagnostic LCD [9]. The authors found that while tablet computers performed inferiorly to the diagnostic LCD when the zoom feature was not enabled, comparable phantom scores were obtained for both displays when zooming was available to the readers. The study also found no reporting discrepancies during the interpretation of 43 normal examinations and 5 cases of acute intracranial hemorrhage and concluded that tablets can be used to identify acute cerebral hemorrhage findings if the software zoom feature is enabled.

Similar results were described by Johnson et al. on a similar experiment comparing radiologists’ interpretative performance of CT images on the tablet to interpretation on a conventional PACS display for pulmonary embolism cases [10]. The authors found that the radiologists interpreted 98% of cases correctly regardless of display platform with no statistically significant difference in sensitivity, specificity, or accuracy of interpretation. This study provides evidence suggesting that CT interpretation might be almost equally accurate when performed using tablet computers as compared to PACS workstation.

In a similar, recent study, Park et al. examined the capability of next-generation tablet computers (iPad 2*^nd^* generation) as a tele-radiology tool for evaluating brain CT images with subtle hemorrhage [11]. The clinician’s performance using the tablet computer was compared to results obtained using a primary-class 2 MP color LCD. The authors reported that the sensitivity and specificity for all clinicians were high and calculated the AUC to find that there was no statistically significant difference between the two display devices. The weighted k values showed moderate to very good intra-observer agreement between the tablet and the LCD. The authors found that clinicians using tablets with a stable internet connection can provide reliable remote evaluation of brain CT images with subtle hemorrhage under sub-optimal viewing conditions.

### Purpose

As these previous studies demonstrate, reader experiments and clinical evaluations point to the potential usefulness of handheld devices in medical imaging applications. However, many of these studies and their comparative findings are limited to specific models. Currently, a number of manufacturers are marketing various types of handheld display devices with different sizes and technological features. Although image quality characteristics of medical workstation displays have been extensively assessed[14]–[17], the rich variety of handheld display devices have not yet been fully characterized. In this paper, we analyze the image quality characteristics in terms of spatial resolution, spatial noise, luminance response, and reflectance for various sized handheld displays including LCDs and organic light-emitting diode (OLED) displays with emissive pixels, thinner designs, more effective power saving, and wider viewing angle performance [18], [19]. We compare the image quality characteristics among a selection of handheld display devices and medical workstation displays and provide an analysis for each display feature based on consistent measurement methodology.

## Materials and Methods

### Displays

We used three phone-sized handhelds: Phone1-LCD (iPhone4, Apple Inc., CA, USA, released in 2010), Phone2-OLED (Nexus One, HTC Corp., Taoyuan, Taiwan, 2010), Phone3-OLED (Galaxy S, Samsung Corp., Seoul, South Korea, 2010), and four tablet-size handhelds: Tablet1-LCD (iPad 1*^st^* generation, Apple Inc., CA, USA, 2010), Tablet2-LCD (Galaxy Note 10.1, Samsung Corp., Seoul, South Korea, 2011), Tablet3-LCD (Galaxy Tab 10.1, Samsung Corp., Seoul, South Korea, 2011), Tablet4-LCD (Nexus 7, ASUSTek Computer Inc., CA, USA, 2012), and Tablet5-LCD (iPad 3*^rd^* generation, Apple Inc., CA, USA, 2012). Phone2-OLED and Phone3-OLED use active-matrix organic light-emitting diode (AMOLED) displays with PenTile sub-pixel technology, and Phone1-LCD, Tablet1-LCD, Tablet4-LCD, and Tablet5-LCD use LCDs with in-plane switching (IPS) technology. The PenTile layout allocates green (G) subpixels interleaved with alternating red (R) and blue (B) subpixels. The R-G-B-G layout is iterated and one pixel is represented by two sub-pixels of R-G or B-G. The G subpixels are mapped on one-to-one correspondence with input signal pixels. The R and B subpixels are sub-sampled and reconstruct the chroma signal. The luminance is processed using adaptive sub-pixel rendering filters from the input image. A 5 MP monochrome workstation LCD: WS-5MPLCD (G51, Eizo Nanao Corp., Ishikawa, Japan) and 3 MP color workstation LCD: WS-3MPLCD (R31, Eizo Nanao Corp., Ishikawa, Japan), which are IPS devices, for primary image diagnosis were used as reference. Table 1 lists the size specifications of all display devices used in this study. We fixed the brightness settings of the handheld displays at the maximum for each device and performed all measurements in a display evaluation laboratory with non-reflective, flat-black walls and controlled lighting.

**Table 1:** Specifications for the display devices tested in this study.

Display	Screen size (inch)	Pixel array	Pixel pitch (mm)
Phone1-LCD	3.5	640×960	0.0780
Phone2-OLED	3.7	480×800	0.101
Phon3-OLED	4.0	480×800	0.109
Tablet1-LCD	9.7	768×1024	0.192
Tablet2-LCD	10	800×1200	0.170
Tablet3-LCD	10	800×1200	0.170
Tablet4-LCD	7.0	800×1200	0.118
Tablet5-LCD	9.7	1536×2048	0.096
WS-5MPLCD	21	2048×2560	0.165
WS-3MPLCD	20	1536×2048	0.207

### Luminance Response

**Figure 1:**
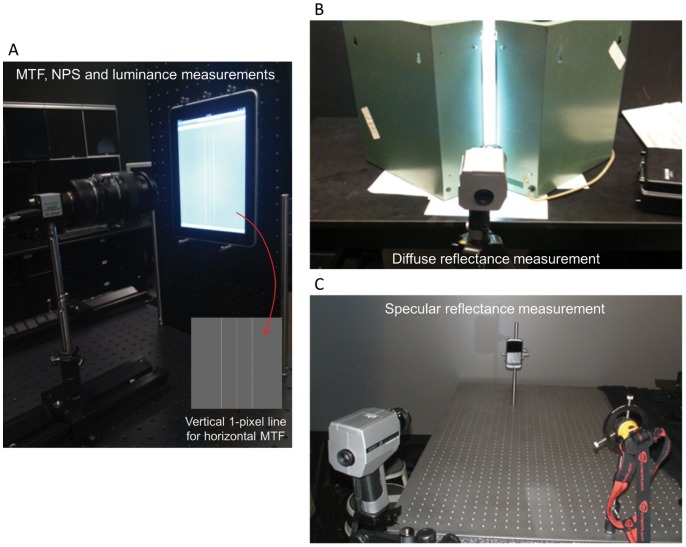
Experiment layouts. **A.** The layout of the photometric CCD camera capturing the display screen for MTF, NPS and luminance measurements. The center line of the vertical 1-pixel line pattern was used for the horizontal LSF and MTF. **B.** The layout of the spot photometer measuring the luminance reflected off the display screen through the gap between the fluorescent lights for diffuse reflectance measurement. **C.** The layout of the spot photometer measuring the luminance reflected off the display using the flash light source for specular reflectance measurement. The photometer and light source were positioned symmetrically 78.7 cm away at 15° from the normal relative to the screen.

Uniform patterns of 18-step digital driving levels (DDLs) (0, 5.88(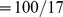)%, 11.8%, 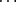, 94.2%, 100%) were displayed respectively and the screen was captured by a photometric charge-coupled device (CCD) camera (P199F, Westboro Photonics Inc., Ottawa, Canada) equipped with a macro lens (NIKON AF Micro-Nikkor 60 mm f/2.8D, Nikon Inc., Tokyo, Japan). The CCD sensors consisted of 1624×1224 elements with 0.0044-mm pixel pitch. Figure 1A shows the experiment layout with the camera capturing the image of the handheld screen. The camera is calibrated at the pixel level to luminance in a range from 0.02 to 50,000 cd/m^2^ with a 12-bit analog-to-digital conversion. Following Ref. [12], the luminance ratio 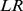 was calculated using minimum and maximum luminance, 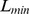 and 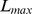 (cd/m^2^), as follows,
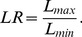
(1)


The luminance data were translated to contrast response 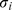 at each luminance step 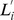 as a function of the mean just-noticeable difference (JND) index 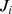 at the step as follows,
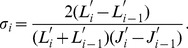
(2)


The expected contrast response 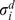 from the Digital Imaging and Communications in Medicine (DICOM) GSDF luminance values was calculated as follows,
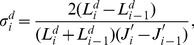
(3)using each luminance 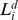 on GSDF corresponding to each step JND index.

### Spatial Resolution

Modulation transfer functions (MTFs) were measured to characterize the spatial resolution of the displays. We used a methodology based on Ref. [12]. We used a pattern image with a horizontal or vertical 1-pixel line in a uniform background (see Fig. 1A). The line and background signal levels were 60% and 50% DDLs, respectively. The displayed pattern was captured with high magnification by the photometric camera. The magnification corresponded to about 9×9 CCD pixels per display pixel. The captured line pattern images were subtracted from the background images, acquired by capturing uniform patterns with the same setup. After subtraction, 1200 line profiles in each captured image were averaged vertically (in the line direction). Line spread functions (LSFs) were calculated by normalizing the averaged profiles by the maximum luminance values. Horizontal and vertical MTFs were calculated by fast Fourier transformation of the LSFs. We represent the MTFs as a function of absolute and relative spatial frequency, with relative spatial frequency being equal to the absolute spatial frequency divided by the Nyquist frequency corresponding to the display system. The MTFs as a function of the relative spatial frequency express how much blur is present regardless of pixel pitch. If there is no resolution degradation on the display, the LSF becomes a square wave and the MTF is given by the sinc function,
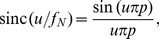
(4)where 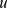 is spatial frequency (mm^−1^), 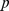 is display-pixel pitch (mm), and 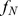 is Nyquist frequency (mm^−1^) = 1/2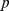.

### Spatial Noise

Noise power spectra (NPS) were measured to characterize the spatial noise following Refs. [12], [14], [16]. A uniform pattern with 50% DDL was displayed and the screen was captured by the photometric camera. For calculating one-dimensional (1D) horizontal NPS, a region of interest (ROI) of width 512 by height 40 pixels, which positioned at the horizontal center and upper end in the captured image, was selected and the 512-point horizontal profile was acquired by averaging the 40 pixel values vertically. This calculation is a numerical-slit method to eliminate vertical noise. After being subtracted from the mean value of 512 data in the averaged profile, the profile was processed with a Hanning window, and fast-Fourier transformed. The window processing works to reduce spectral leakage errors [16] in Fourier space, particularly since displays have periodic pixel structures inducing spectral peaks at frequencies in accordance with the integral multiples of the inverse of the sub-pixel pitch. The 1D 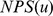 (mm^2^) calculation is expressed as follows,
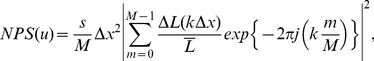
(5)with 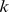 = 0, 1, 2, 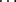, 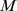-1, where 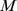 is noise profile data points 512, 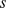 is numerical-slit length points 40, 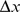 is effective camera-pixel pitch (mm) on captured plane, 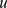 = 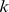/(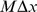) is spatial frequency (mm^−1^), 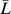 is average luminance (cd/m^2^) of 512 data, 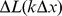 is luminance difference (cd/m^2^) at 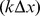 from 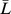. The 512×40 ROI was moved vertically without overlaps to repeat the NPS calculation as many times as possible and the NPS were averaged. Vertical NPS was calculated in the same way using the horizontal numerical-slit scanning. Furthermore, two-dimensional (2D) 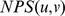 (mm^2^) was calculated by a 2D fast Fourier transformation with a Hanning window processing as follows,
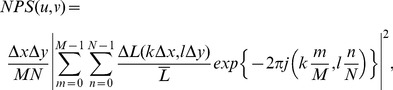
(6)with 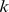 = 0, 1, 2, 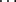, 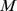-1, and 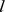 = 0, 1, 2, 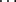, 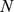-1, where 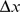 and 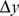 are effective camera-pixel pitches (mm) on captured plane, 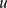 = 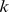/(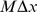) and 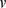 = 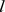/(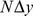) are spatial frequencies (mm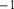), 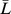 is average luminance (cd/m2) in ROI with 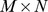: 256×256 matrices, 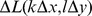 is luminance difference (cd/m2) at 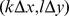 (cd/m2) from 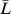.

### Reflectance

Display reflectance was characterized by specular and diffuse components of the overall reflection coefficient. We measured the diffuse and specular reflection coefficients for each display following methods described in Ref. [12], [20]. Each handheld display was turned off and placed on the back of a white styrofoam box. The back of the box is removable to measure large medical workstation displays. Subsequently, two fluorescent lamps with light diffusers were symmetrically positioned toward the inside of the box at the opening with a small gap between them (see Fig. 1B). An illuminance meter (T-10, Konica Minolta Sensing, Inc., Tokyo, Japan) was placed into the Styrofoam box to measure the illuminance inside the box at the screen surface. The box was closed off with the fluorescent lights and the luminance of the diffuse reflection off the screen was measured using a spot photometer (CS-100, Konica Minolta Sensing, Inc., Tokyo, Japan) through a gap between the fluorescent lights. The diffuse reflectance coefficient 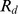 (cd/m^2^/lx), was calculated as follows,
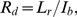
(7)where 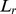 is the reflected luminance (cd/m^2^) measured with the spot photometer and 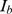 is the illuminance (lx) at the surface of the displays when the illuminating box is on. The illuminance 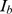 inside the Styrofoam box was approximately 5,280 lx.

To measure the specular reflection coefficient, a 7-LED flash light source was fixed 78.7 cm away from the device at an angle of 15° from the normal relative to the center of the screen, pointing at the center of the handheld display. Symmetrically, the spot photometer was fixed 78.7 cm away at 15° from the normal relative to the screen (see Fig. 1C). The photometer measured the reflected luminance from the flash light source. Next, the photometer was placed 157.5 cm away from the LED light and measured the direct-view luminance. The specular reflectance coefficient 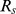 was calculated as follows,
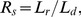
(8)where 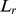 and 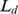 are the measured luminance values (cd/m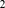) from the reflection and from the direct view, respectively.

## Results and Discussion

**Figure 2:**
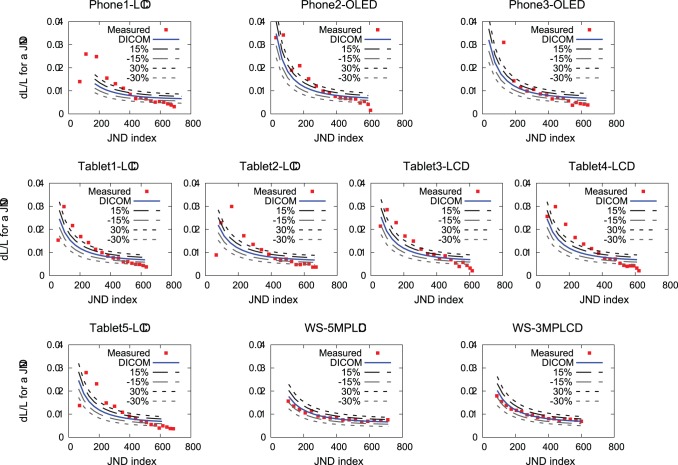
Contrast responses at 18 luminance levels as a function of JND index. The measured contrast responses at each step and the expected contrast responses from DICOM GSDF with the 15 and 30% tolerance bands are depicted.

Table 2 shows the minimum and maximum luminance values and the luminance ratios as the handheld display brightness settings are fixed at maximum. WS-5MPLCD and WS-3MPLCD have relatively high minimum luminance and low luminance ratios. Phone2-OLED and Phone3-OLED have lower minimum luminance than others and the luminance ratios are noticeably higher. The maximum luminance of WS-3MPLCD is the lowest and it results in the lowest luminance ratio. The luminance ratios of all handheld displays are higher than those of WS-5MPLCD and WS-3MPLCD. Figure 2 shows the measured contrast responses 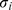 at the 18-step luminance levels and the expected contrast responses 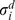 from DICOM GSDF as a function of the JND with 15% and 30% tolerance bands. While the differences 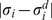 for WS-5MPLCD and WS-3MPLCD are within the 30% tolerance bands in all JND index ranges, all handheld displays have 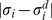 beyond the 30% tolerance bands.

**Table 2:** Minimum and maximum luminance values and luminance ratios for the devices tested in this study.

Display	*L_min_*	*L_max_*	*LR*
Phone1-LCD	0.770	479	622
Phoe2-OLED	0.138	259	1886
Phoe3-OLED	0.174	341	1962
Tablet1-LCD	0.511	328	642
Tablet2-LCD	0.737	426	579
Tablet3-LCD	0.471	280	595
Tablet4-LCD	0.521	295	566
Tablet5-LCD	0.750	467	623
WS-5MPLCD	1.51	595	395
WS-3MPLCD	1.05	271	258

The unit for luminance is cd/m^2^.

We observe that AMOLED displays have higher luminance ratios due to the low minimum luminance values compared to LCDs. We also found that WS-5MPLCD and WS-3MPLCD had low luminance ratios compared to all handheld displays since the medical workstation displays were calibrated to comply with GSDF. Accordingly, the contrast response values of the workstation displays are within the 30% tolerance limit. On the other hand, the contrast response of all handheld displays exceed the 30% tolerance limit. Image contrast is one of the important factors to determine performance for clinical diagnosis. This device dependency of the luminance response could lead to inconsistent clinical decisions. In addition, all of our measurements are performed with handheld displays at the maximum brightness settings. The results of our analysis could significantly vary if other manually selected brightness level or the auto-brightness setting was used.

**Figure 3:**
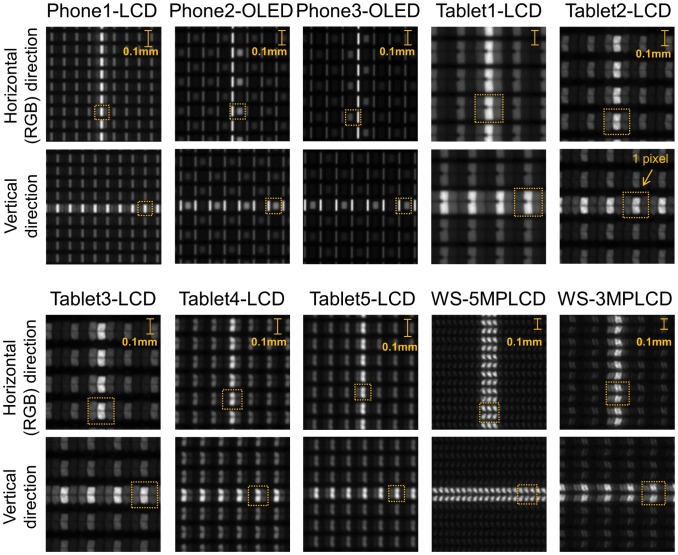
Display pixel structure. The screen displaying the 1-pixel line pattern was captured by the photometric camera. Since these image are not exactly in the same scale, 0.1-mm scale bars are indicated. Squares bounded by orange dot lines show the one pixel region of the displays. The vertical lines reflect the horizontal resolution characteristics corresponding to the RGB direction.

**Figure 4:**
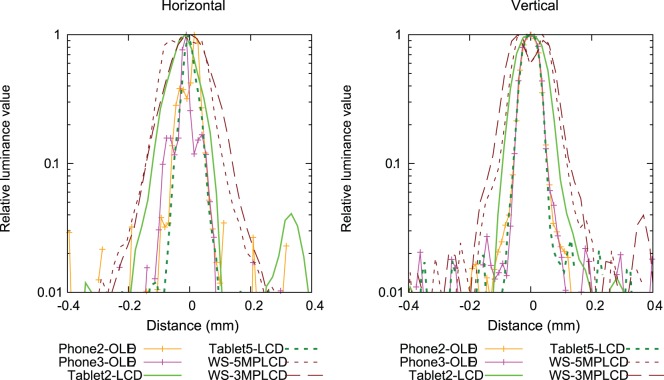
Line spread functions for the devices tested in this study. The relative luminance profiles on a 1-pixel line are shown on logarithmic scale.

Figure 3 shows the captured images displaying the 1-pixel line on each display. We denote red-green-blue (RGB) direction of subpixels as the horizontal direction. The vertical line images reflect the horizontal resolution characteristics. The sub-pixel shapes and layouts on the Phone2-OLED and Phone3-OLED with PenTile technology are different from those seen on LCD devices. When the vertical line is displayed on the Phone2-OLED, the illuminated red or blue sub-pixel is located on only one side of the green sub-pixel. In the case of Phone3-OLED, the red or blue subpixels on both sides of green are illuminated. The illuminated red or blue sub-pixel location is determined by the respective sub-pixel rendering algorithms. The line profiles of some displays are represented using a logarithmic scale in Fig. 4. The LSFs have negative values because of the subtraction from the background images. WS-5MPLCD, the only monochrome display in this study, have each sub-pixel with almost same luminance values and the LSF is the closest to a square pattern. On the other hand, green subpixels of other color displays have prominently higher luminance values than red and blue sub-pixel luminance values and the LSFs are narrower than for WS-5MPLCD.

**Figure 5:**
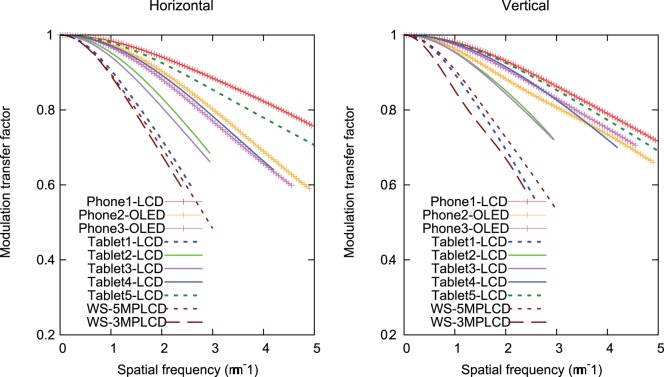
Modulation transfer functions as a function of absolute spatial frequency for the devices tested in this study.

**Figure 6:**
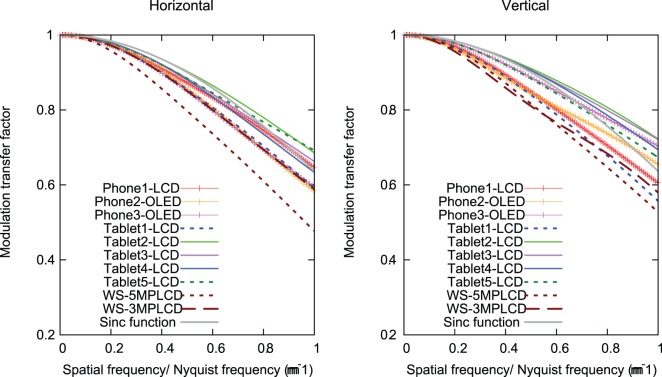
Modulation transfer functions as a function of relative spatial frequency for the devices tested in this study. Sinc function representing the ideal MTF is included.

Figure 5 shows MTFs as a function of absolute frequency in the horizontal and vertical directions for all displays. The displays with smaller pixel pitch tend to have higher MTFs. The Tablet1-LCD and WS-3MPLCD have lower MTFs due to their larger pixel pitch. Although the pixel pitch of the WS-5MPLCD is smaller than the pitch of the WS-3MPLCD, the both MTFs show similar values in horizontal direction, because each sub-pixel in one pixel on the monochrome WS-5MPLCD exhibits almost same luminance resulting in the LSF close to a square wave. The MTF as a function of relative spatial frequency in Fig. 6 allows for comparisons of the spatial resolution regardless of pixel pitch. Tablet2-LCD has the highest MTFs in both horizontal and vertical directions, followed by Tablet3-LCD, Tablet4-LCD, and Tablet5-LCD, and their MTFs are closer to the sinc function expressing the ideal MTF. In the high frequency range, their MTFs exceed the sinc function. The vertical MTFs of Phone2-OLED and Phone3-OLED get close to the sinc function in the high frequency range.

While the sub-pixel rendering in Phone2-OLED results in asymmetric horizontal LSF, the resolution characteristics show no significant differences as seen in the MTF as a function of relative spatial frequency. The monochrome WS-5MPLCD shows an horizontal LSF closer to a square pattern and correspondingly lower MTF compared to other color display MTFs with smaller pixel pitch. This is due in part to subpixels in the monochrome display exhibiting almost uniform luminance for the line pattern. As evidenced by Figs. 3 and 4, all color displays exhibit higher luminance in the green sub-pixel compared to the red and blue sub-pixel luminance with narrower LSFs compared to a square pattern. We found that the tablet LCDs released after 2011 have comparable resolution characteristics to the ideal display, while Tablet1-LCD released in 2010 has slightly degraded sharpness compared to the recent devices. The AMOLED mobile phones have resolution characteristics close to the ideal display in the vertical direction due to the PenTile sub-pixel layout and rendering algorithm.

**Figure 7:**
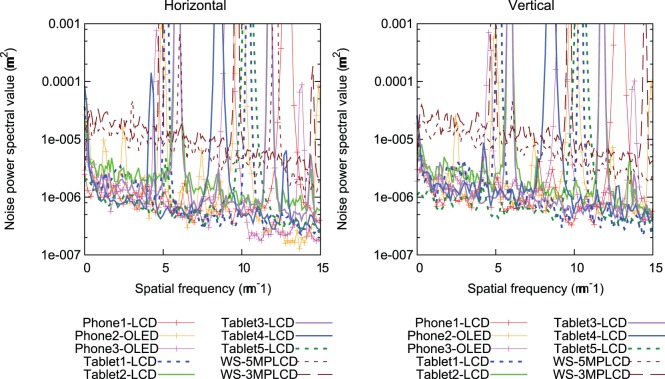
One-dimensional noise power spectra for the devices tested in this study.

**Figure 8:**
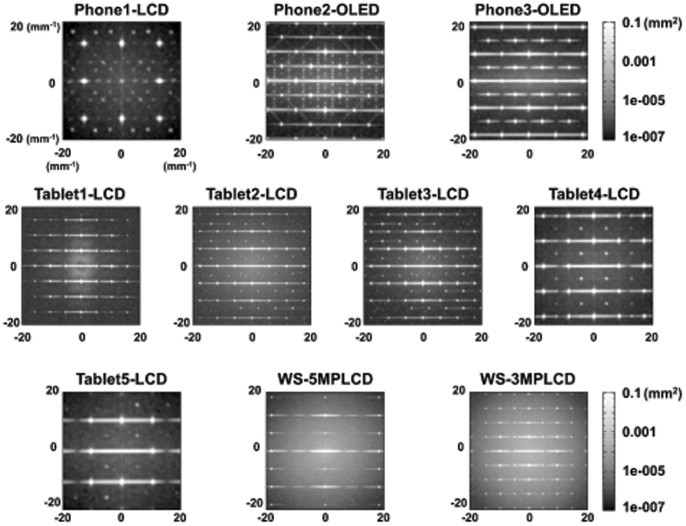
Two-dimensional noise power spectra for the devices tested in this study.

Figure 7 shows 1D NPS in the horizontal and vertical directions for all displays. In both directions, WS-5MPLCD and WS-3MPLCD have approximately 10 times higher NPS values than all handheld display devices. The 2D NPS shown in Fig. 8 depict worse noise characteristics corresponding to WS-5MPLCD and WS-3MPLCD compared to the handheld display characteristics, which are emphasized at larger gray levels. The noise characteristic advantages in the handheld displays would contribute to low-contrast detectability.

All of the handheld displays have superior or comparable spatial resolution and noise characteristics especially for the recent generation devices to the medical workstation display characteristics. However, the metrics do not include image processing effects, since the MTFs and NPS just evaluate the display inherent physical characteristics. In practical applications, images with a large number of pixels would be displayed with a reduced magnification. The compression processing, which usually calculates pixel values using re-sampling, could cause aliasing errors and further deteriorate spatial resolution. Smoothing processing is often used to suppress aliasing errors, but it does not avoid degrading the resolution. The resolution degradation is more remarkable at more reduced magnifications for displays with smaller screens. In addition, the observed object sizes are adjustable based on viewing distance. The medical workstation displays are generally assumed to be observed with longer viewing distance (30 cm) than the distance for handheld viewing. Hence, the resolution characteristics of the workstation displays at the observed distance should be better estimated by MTFs shifted to higher frequency. We cannot conclude that the displayed image quality characteristics on all handheld displays are better than those on the workstation displays based on the MTF measurements. An analysis that takes into account image processing effects and actual size would be needed to evaluate overall image quality.

**Figure 9:**
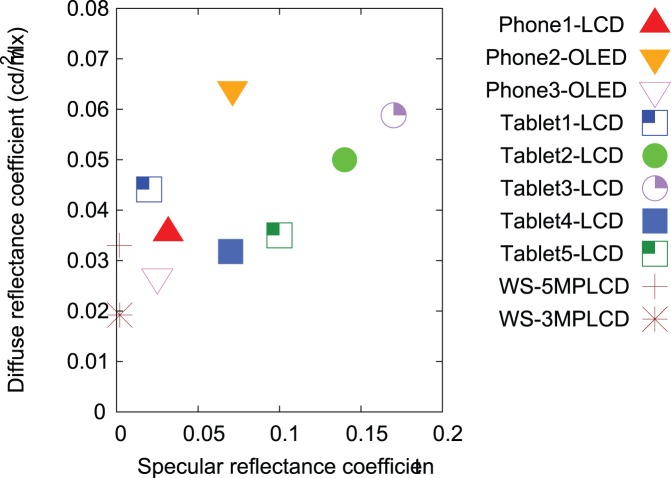
Diffuse reflectance coefficients as a function of specular reflectance coefficients for the devices tested in this study.

Figure 9 shows the diffuse reflectance coefficients 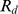 as a function of the specular reflectance coefficients 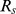. While WS-5MPLCD and WS-3MPLCD have 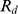 0.0017 and 0.0020, respectively, all handheld displays show higher 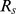 than the workstation displays. Especially, Phone2-OLED has the highest diffuse reflectance 0.064 among all displays, and Tablet2-LCD and Tablet3-LCD have high reflectance coefficients for both 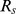 and 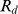. Phone1-LCD and Phone3-OLED have relatively similar reflectance coefficients to the workstation displays for both 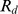 and 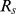.

The reflectance results suggest that Phone2-OLED, Tablet2-LCD, and Tablet3-LCD might suffer more in terms of image quality at higher illumination environments due to their high diffuse and specular reflectance that induces glare and deteriorates viewing performance. Conversely, the reflectance of Phone1-LCD, Phone3-OLED are lower, closer to those of medical workstation displays making these handheld displays potentially more appropriate for image viewing over a wider range of ambient illumination conditions. However, more studies are needed to validate our findings in ambient illumination scenarios including the effects of light source spectral content and angular distribution.

Initial work on quantifying the performance of mobile display devices with applications in medical imaging reported by Vogel et al. [21] centered on assessing the performance of human and computational observers for a detection task using digitally inserted target on flat backgrounds. Ultimately, the present study needs to be complemented with an investigation into how close different aspects of image quality analyzed in this work correlate with diagnostic performance for the specific visual task involved in the imaging modality being tested.

## Conclusions

We prove that handheld displays can have improved spatial resolution and noise characteristics compared to medical workstation displays particularly for recent hardware of the devices. However, since the luminance characteristics of handheld displays do not comply with the GSDF response, the displayed image contrast is different from images radiologists and medical staff are familiar with viewing on their workstation displays. When compared to medical workstation displays, some handheld displays exhibit similar diffuse and specular reflectance properties, but in most cases, those of handhelds are much higher. In summary, the results demonstrate that handheld displays can have good image quality characteristics compared to medical workstation displays in terms of spatial resolution and noise and reflectance. However, other factors that affect the quality of the displayed image need to be considered including image size and ambient illumination for comprehensive image quality estimation. Further investigations that take into account such factors lead to conclude whether handheld devices might provide a reliable image viewing platform for medical imaging applications.
